# Survey on awareness and preference of ceramic bracket debonding techniques among orthodontists

**DOI:** 10.4317/jced.56976

**Published:** 2020-07-01

**Authors:** Aileen Y. Ngan, Prashanti Bollu, Kishore Chaudhry, Richard Stevens, Karthikeyan Subramani

**Affiliations:** 1 Roseman University of Health Sciences, College of Dental Medicine, Henderson, NV, USA

## Abstract

**Background:**

The objectives of this study was to evaluate the awareness of different ceramic bracket debonding techniques among orthodontists in the USA and the most commonly used debonding technique for ceramic bracket removal.

**Material and Methods:**

A survey on preference for debonding and awareness of debonding techniques was emailed to 2,227 members of the American Association of Orthodontists (AAO).

**Results:**

119 orthodontists completed the survey. 111 responses were included in the study analysis of ceramic bracket users. The most common technique used was mechanical debonding. 86.5% used a specially designed bracket removing plier from the manufacturer. Overall, there were 59.5% of surveyed orthodontists who were aware of electrothermal debonding, 73% were unaware of ultrasonic debonding and 83.8% were unaware of laser debonding. There were more orthodontists with an affiliation with an academic institution aware of electrothermal debonding (*p*=0.002). There also was a trend of orthodontists having no affiliation with an institution who were unaware of laser debonding (*p*=0.015).

**Conclusions:**

This survey showed that the majority of orthodontists who responded to the questionnaire were unaware of alternative debonding techniques of ceramic brackets. All orthodontists who use ceramic brackets utilized mechanical debonding technique.

** Key words:**Orthodontic ceramic brackets, mechanical, electrothermal, ultrasonic, laser debonding.

## Introduction

Clear brackets have become largely sought after due to increasing number of adult patients seeking orthodontic treatment with esthetic needs (1). There are different types of clear brackets to meet the demand for esthetic treatment. Plastic brackets provide improved esthetics, however they present a few clinical problems. Feldner *et al.* (2) showed that pure plastic polycarbonate brackets appeared to have higher deformation values than metal brackets. In the 1970’s, composite resin brackets were developed as an alternative to metal brackets. However these brackets lost popularity because of fracture, discoloration, deformation, slot distortion caused by water absorption and lower torque expression (3). In 1987, ceramic brackets were introduced, despite a lack of clinical and scientific data regarding their bonding and debonding characteristics (4).

There are two types of ceramic brackets: monocrystalline and polycrystalline. The effect of two different manufacturing processes results in different physical characteristics of monocrystalline and polycrystalline brackets. Polycrystalline ceramic brackets are sintered or fused aluminum oxide particles (1). Slight imperfections and impurities from sintering can serve as foci for crack propagation under stress (15). In contrast, the oxide particles in monocrystalline ceramic brackets undergo crystallization and are milled into shape. However when the monocrystalline bracket is scratched, the fracture resistance decreases drastically (5). The ability to resist structural failure is much stronger in monocrystalline than polycrystalline (4).

Ceramic brackets are composed of aluminum oxide particles (1). Aluminum oxide is an inert material which cannot chemically adhere directly to bonding resins. Therefore bonding of ceramic brackets is accomplished by incorporation of a silane coupling agent into the bracket design by manufacturers, mechanical retention, or combination of both (4,6,7). This results in higher bond strength between the bracket-adhesive interface which can result in increased stress to the enamel-adhesive interface during debonding (3,8). Although ceramic brackets are esthetic, enamel fractures and bracket fractures frequently occur during conventional debonding procedures (9).

The “fracture toughness” (or the ability of a material to resist fracture) of ceramic brackets is much lower than stainless steel (7). Debonding pressure on the bracket base often results in partial or complete bracket failure or fracture. The removal of the remaining fragments has to be carried out with a diamond bur which can become a time-consuming procedure (9). The potential for both enamel and bracket fracture has created a need for safer and more reliable method of debonding ceramic brackets (7,10).

Manufactures and researchers have been continuously working to modify the base design of ceramic brackets and develop specially designed mechanical debonding instruments (1,8). Chen *et al.* (8) showed that new designs with a ball reduction band in the Inspire Ice bracket (Ormco, Orange, CA) and vertical debonding slot in the Clarity bracket (3M Unitek, Monrovia, CA) reduced the risk of ceramic bracket fracture during debonding. Even then, enamel damage regularly occurred with ceramic brackets compared with metal brackets (11).

Alternative debonding techniques that minimize bracket failure as well as trauma to the enamel surface have been studied (7). Ultrasonic debonding technique uses specially designed tips to apply vibrations at the bracket-adhesive interface to erode the adhesive layer between the enamel surface and bracket base (7). Boyer *et al.* (12) found that the ultrasonic chisel markedly reduced debonding force. However, debonding time was significantly greater than conventional methods and excessive wear of the expensive ultrasonic tips occurred (12,13).

Electrothermal debonding was first described by Sheridan *et al.* (14) for removing metal brackets from enamel by heat generated from a cordless battery device. Heat is transferred to the bracket by a blade that is placed in the bracket slot (7,14). When the heat applied to the bracket is transferred to the adhesive and deforms the adhesive-bracket interface, the bracket can be lifted from the enamel surface without distortion of the bracket or excessive force to the underlying enamel (14). Electrothermal technique was found to be quick, effective, and devoid of either bracket or enamel fracture. However this method raises concern on potential pulp damage because of a significant rise in pulpal temperature (13,15).

The most recent alternative debonding technique is the use of lasers. Laser-aided debonding of ceramic brackets is conceptually similar to the use of the electrothermal approach by heat generation to soften the adhesive. Adhesive softening occurs through three processes: thermal softening, thermal ablation and photoablation (10). The process of thermal softening occurs when the bonding agent is heated and the bracket slides off the tooth surface. Thermal ablation is the process whereby the temperature increases rapidly in an adhesive resin vaporization range and the bracket blows off the tooth surface. In photoablation, the energy level of the bonds between the bonding-resin atoms rapidly increases above their dissociation energy levels resulting in decomposition of the material (10).

There are 4 major types of lasers classified by their lasing mediums: gas, liquid, solid, and semiconductor (or laser diode). Various studies have been conducted to explore the applicability of lasers for ceramic bracket removal. Ghazanfari *et al.* (10) reported that laser irradiation is an efficient way to reduce shear bond strength of ceramic bracket and debonding time. However, a major concern is the potential for thermal irritation of the pulp caused by inaccurate method and duration of laser pulse (9,10,16). Macri *et al.* (16) found that CO2 laser decreased the bond strength without increasing temperature excessively. Sarp *et al.* (9) used a 1,070-nm Ytterbium fiber laser to debond ceramic brackets and found decreased bond strength, debonding time, and work done with minimal intrapulpal temperature change.

With the advent of studies on the alternative debonding techniques of ceramic brackets, there lacks information in the literature on the most used method of debonding ceramic brackets as well as the awareness of alternative debonding techniques amongst orthodontists. The purpose of this study was to conduct a survey on the awareness level and preference of debonding techniques of ceramic brackets amongst orthodontists.

## Material and Methods

A survey questionnaire was developed with an online survey platform (www.qualtrics.com). This study was approved by the Roseman University of Health Sciences Institutional Review Board. The survey questionnaire was reviewed and approved by American Association of Orthodontists (AAO) Partners in Research. Data was collected over 45 days with an email sent to 2,227 members of the AAO. A reminder email was sent after 30 days and the survey remained open for another 15 days. A cover letter discussing the aims of the study and a link to the survey of questions was included in the email. Retired orthodontists and orthodontic resident members of the AAO were excluded from the study.

All data collected over 45 days was analyzed with IBM® SPSS® version 25. Descriptive statistics was calculated using chi-square tests. Frequencies determined number of clinicians who use ceramic brackets and most used debonding technique. Possible contributing factors for level of awareness on debonding techniques were further analyzed.

## Results

A total of 119 clinicians completed the survey, representing a response rate of 5.34% of the interviewed population. There were six clinicians who do not use ceramic brackets and were therefore excluded from analysis. Additionally, two responses were removed from analysis because of inconsistency in their responses. Those two respondents were excluded because they were unaware of mechanical, debonding technique despite choosing answer choices that indicated that they were utilizing mechanical debonding.

**Figure 1:**
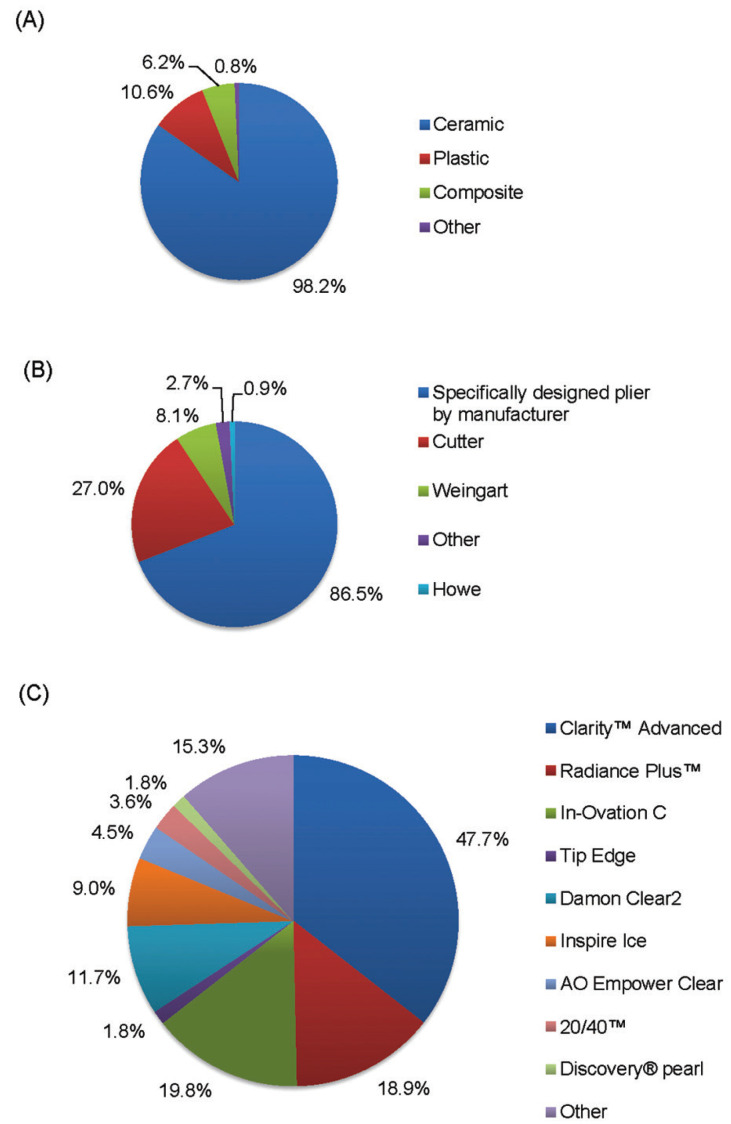
Pie charts depicting the percentage of (A) ceramic bracket users amongst orthodontists who completed the survey, (B) most commonly used mechanical debonding plier, and (C) percentage of various brands of ceramic brackets used by orthodontists.

Out of the 111 orthodontists who have experience with clear brackets in practice 98.2% use ceramic, 6.2% use composite, 10.6% use plastic, and 0.8% use an unspecified clear bracket (Fig. 1A). All of the respondents who have used ceramic brackets use mechanical debonding. Majority of clinicians (86.5%) debond ceramic brackets with the manufacturer’s specific bracket removing plier (Fig. 1B). The most common ceramic bracket used is the 3M Clarity Advanced (47.7%) ceramic bracket (Fig. 1C).

When asked if debonding of ceramic brackets raised any level of concern for potential side effects, 55.9% had minimal concern level and 44.1% had moderate to high level of concerns. Amongst those with moderate to high levels of concern, 17 clinicians have seen less than 10% of their cases with enamel damages and 4 clinicians have seen approximately 10-30% of their cases with enamel damages (Table 1).

**Table 1:**
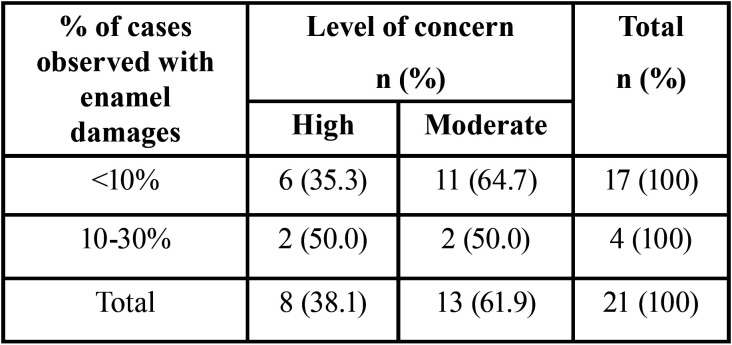
Percentage of cases with enamel damage amongst orthodontists with moderate to high levels of concern for side effects with debonding ceramic brackets.

Table 2 compares orthodontists’ years of experience in practice and their level of awareness on the various debonding techniques of ceramic brackets. Column of unaware for mechanical debonding and column of very aware for laser debonding was excluded from chi square calculation because none of the respondents selected those answer choices. All orthodontists were aware of mechanical debonding. There was a correlation between years of experience and awareness level for mechanical debonding (*p* = 0.013). Majority of orthodontists (83.8%) were unaware of laser debonding of ceramic brackets compared to the other debonding methods. Additionally, majority of orthodontists were unaware of ultrasonic debonding (72.9%) and unaware of electrothermal debonding (59.5%). 47.2% of orthodontists with 15+ years of experience were very aware or somewhat aware and 52.8% of orthodontists with 15+ years of experience were unaware of electrothermal debonding.

**Table 2:**
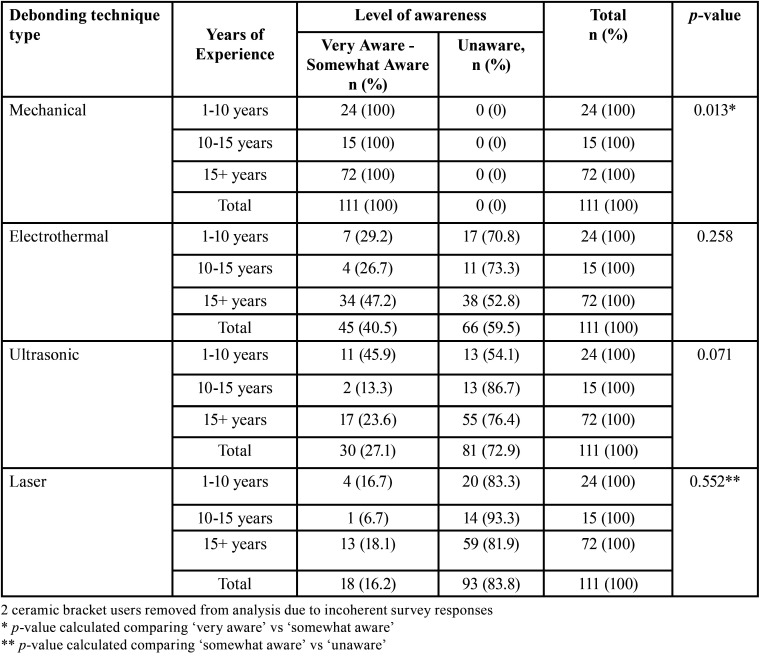
Level of awareness of mechanical, electrothermal, ultrasonic, and laser debonding techniques of ceramic brackets. Orthodontists’ years of experience in practice to level of awareness. Years of experience has been categorized into three groups: 1-10 years, 10-15 years, and 15+ years.

Table 3 compares an orthodontist’s affiliation with an academic institution and their level of awareness on the various debonding techniques of ceramic brackets. Majority of orthodontists with no affiliation with an academic institution were unaware of electrothermal (71.3%), ultrasonic (76.3%), and laser (90.0%) debonding. There was a trend towards the orthodontist’s affiliation with an academic institution and awareness level of electrothermal debonding (*p* = 0.002) and laser debonding (*p* = 0.015).

**Table 3:**
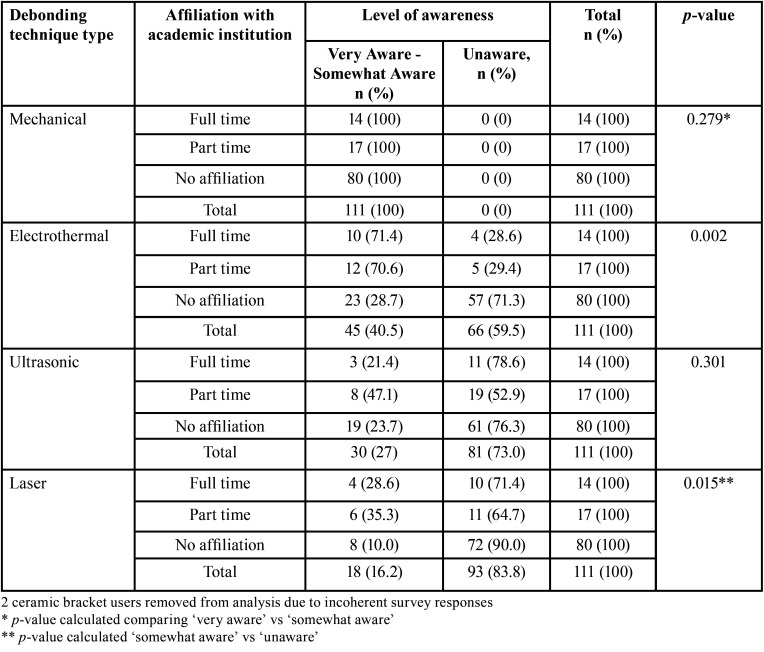
Level of awareness of mechanical, electrothermal, ultrasonic, and laser debonding techniques of ceramic brackets. Orthodontists affiliated to academic institution and their level of awareness. Affiliation with academia has been categorized into three groups: full time, part time, and no affiliation.

## Discussion

In a recent study, Sfondrini *et al.* (17) conducted a survey on orthodontic debonding techniques to find the most commonly used methods to remove brackets and adhesive from tooth surfaces. The results showed that the most commonly used pliers for bracket debonding were the cutter and bracket removal pliers. The study concluded high variability of different methods for bracket debonding, adhesive removal, and tooth polishing. However, the study did not distinguish preferences for debonding techniques of metal and ceramic brackets.

Our study found that all of the orthodontists with clinical practice of ceramic brackets use mechanical debonding and majority were unaware of alternative debonding techniques (ultrasonic, electrothermal and laser). The most commonly used mechanical debonding plier was the manufacturer’s recommended bracket remover plier followed by ligature cutter plier (Fig. 1B) similar to previous survey studies (17,18). Even though mechanical debonding was the preferred method of removing ceramic brackets, many orthodontists (73.9%) responded that they would consider an alternative method. The stronger the bond strength between ceramic bracket and the enamel, the more critical it is to consider alternative methods for bracket removal (1).

Participants were asked to assess their level of concerns for potential side effects with debonding of ceramic brackets and majority (62%) had low concerns. Concerns listed included enamel loss, patient discomfort, bracket and tie wing fracture, amount of remnant cleanup, and patient and practitioner injuries. However those with moderate to high levels of concern reported approximately less than 30% of their debonded cases with enamel damages (Table 1).

When ceramic brackets were first introduced into the market, the bond strength between bonding adhesive, silane coupler, and alumina oxide crystal of ceramic brackets resulted in greater risk of bracket failure and potential for damage during mechanical debonding (19). Moisture, temperature, and other oral variables are known to weaken bond strength at the enamel-adhesive interface (1). Use of an electrothermal machine to assist in debonding ceramic brackets was developed during the early introduction of ceramic brackets. In 1991 Kraut *et al.* (19) determined that the Dentaurum thermal debonding unit was an effective, atraumatic, and physiologically accepTable alternative to mechanical bracket removal in both laboratory and clinical testing (19). Although pulp changes were fairly mild, there was a need for further investigations on the effects of this procedure.

**Figure 2:**
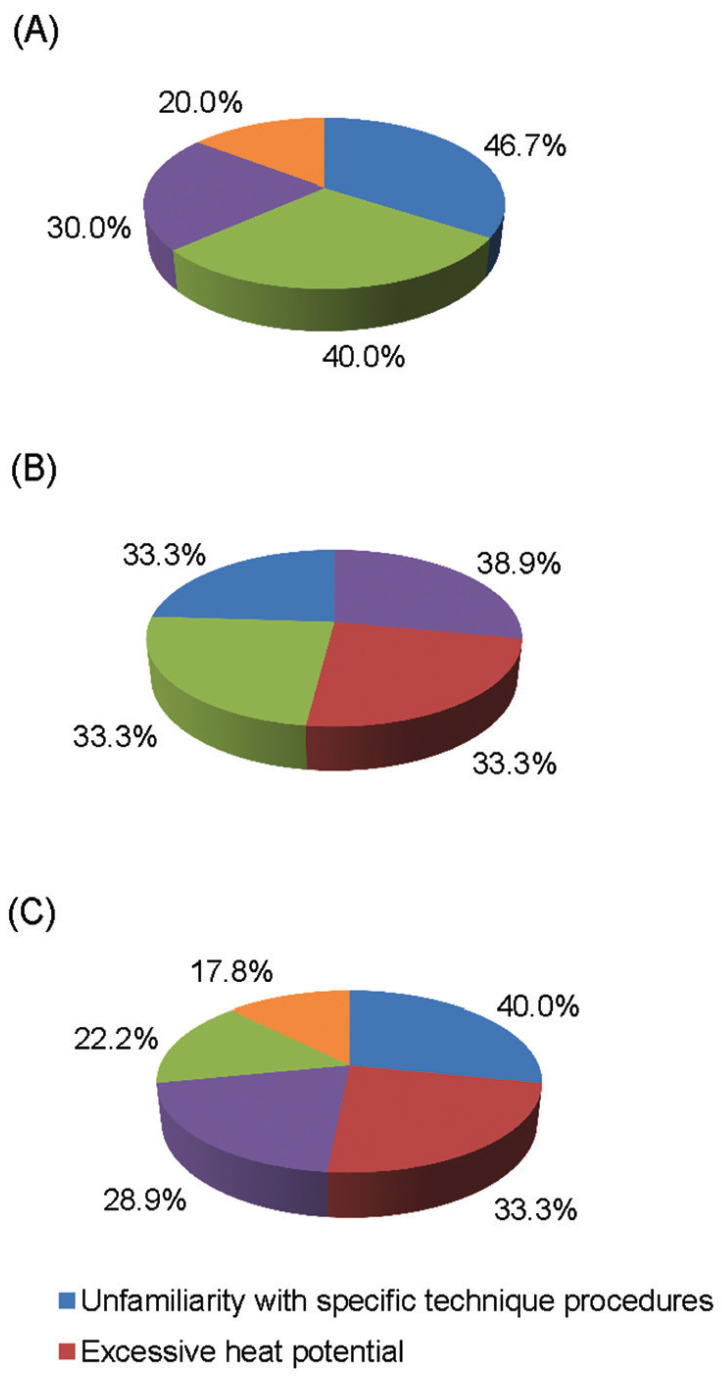
Clinical and practice management reasons why orthodontists choose not to use alternative debonding techniques like (A) Ultrasonic (B) Laser, and (C) Electrothermal.

In this study, of the clinicians who were aware of electrothermal debonding, chose not to use this method because of high costs of the machine, time to debond, excessive heat potential, previous experience, and unfamiliarity with technique (Fig. 2). As shown in Table 3, the clinicians who were aware of electrothermal debonding, had some affiliation with an academic institution. However majority of the orthodontists remain unaware of electrothermal debonding.

Along with the clinical research on electrothermal orthodontic bracket removal, ultrasonic debonding was studied to aid in decreasing bracket adhesive bond strength of ceramic brackets (1,13). However, majority of the respondents were unaware of ultrasonic debonding of ceramic brackets. There was no correlation between years of experience and affiliation with an institution to the level of awareness of ultrasonic debonding. Those who were aware of ultrasonic debonding selected the time to debond, cost of machine, unfamiliarity with technique, and previous experience as reasons for not using this debonding method (Fig. 2). The study by Boyer *et al.* (12) showed that the ultrasonic debonding technique reduced the forces to remove brackets but would be uncomforTable for patient given the extra time required to debond.

The laser approach is more precise with regard to time and amount of heat application however cost of the instrument is high (1). In this study ( Table 2, Table 3), majority of orthodontists are unaware of laser debonding of ceramic brackets. Those aware of laser debonding of ceramic brackets chose not to use because of cost, excessive heat potential, and unfamiliarity with technique. As thermal pulpal irritation is possible during laser-aided debonding, the method and duration of the laser pulse must be exactly defined according to the adhesive resin type (10). Another important consideration in pulpal temperature increase is the type of bracket to be removed. Ivanov *et al.* (10) used a diode laser and found that polycrystalline brackets was cooler than monocrystalline brackets.

Although these methods can be used successfully to debond brackets, the use of pliers is perhaps the most convenient and continues to be the most popular method used for debonding brackets (3). The varying levels of awareness of mechanical debonding ( Table 1, Table 2), highlight several components involved in mechanical debonding of ceramic brackets. Choudhary *et al.* (3) demonstrated that debonding efficiency of a specially designed instrument for ceramic debonding was better than conventional debonding pliers. Additionally, if the adhesive removal technique applies predominantly compressive forces to the enamel, then it can better withstand the trauma compared with tensile forces that can generate cracks and tearouts (11). Another logical approach to reduce force transmission to enamel is to use a bonding material such as resin-modified glass ionomer that is weaker cohesively or has reduced bond strength to enamel (11).

The study by Sfondrini *et al.* (17) found 89.14% of their subjects used metal brackets and 1.5% used ceramic brackets. However our study did not find the percentage of ceramic bracket cases practiced by the respondents. This may have affected the number of orthodontists who were somewhat aware and very aware of mechanical debonding of ceramic brackets (Table 2). It is plausible that orthodontists who were somewhat aware do not practice ceramic brackets as frequently as those orthodontists who were very aware of risks and attributes of ceramic bracket debonding.

Why were the majority of the orthodontists unaware of nonconventional debonding techniques? In the beginning of the introduction of ceramic brackets, enamel fractures, cracks, and flaking had been reported as complications of mechanical debonding (20). These problems led to the investigation of other various debonding techniques to mitigate those side effects. A better understanding of the characteristics of ceramics, enamel, the bond strength of various adhesive systems, and the methods of bracket removal should have assisted the manufacturers in developing a more reliable and clinically safer ceramic bracket (20). In response to the progress of ceramic brackets, Bishara justly emphasized that the development of national/international standards would be useful to manufacturers as well as clinicians, which would help us better serve our patients (20).

## Conclusions

•Mechanical debonding was the most preferred method of ceramic bracket debonding among orthodontists who responded to this survey study.

• Majority of orthodontists who responded to this survey study were unaware of alternative debonding techniques of ceramic brackets.
